# Lower Funneling Pathways in *Scedosporium* Species

**DOI:** 10.3389/fmicb.2021.630753

**Published:** 2021-07-02

**Authors:** Wilfried Poirier, Kevin Ravenel, Jean-Philippe Bouchara, Sandrine Giraud

**Affiliations:** UNIV Angers, UNIV Brest, Groupe d’Etude des Interactions Hôte-Pathogène (GEIHP), SFR ICAT, Angers, France

**Keywords:** lignin degradation, central aromatic molecules, *Scedosporium*, gene cluster, catechol, hydroxyquinol, gentisate

## Abstract

Lignin, a natural polyaromatic macromolecule, represents an essential component of the lignocellulose biomass. Due to its complexity, the natural degradation of this molecule by microorganisms still remains largely misunderstood. Extracellular oxidative degradation is followed by intracellular metabolic degradation of conserved aromatic intermediate compounds (protocatechuate, catechol, hydroxyquinol, and gentisic acid) that are used as carbon and energy sources. The lower funneling pathways are characterized by the opening of the aromatic ring of these molecules through dioxygenases, leading to degradation products that finally enter into the tricarboxylic acid (TCA) cycle. In order to better understand the adaptation mechanisms of *Scedosporium* species to their environment, these specific catabolism pathways were studied. Genes encoding ring-cleaving dioxygenases were identified in *Scedosporium* genomes by sequence homology, and a bioinformatic analysis of the organization of the corresponding gene clusters was performed. In addition, these predictions were confirmed by evaluation of the expression level of the genes of the gentisic acid cluster. When the fungus was cultivated in the presence of lignin or gentisic acid as sole carbon source, experiments revealed that the genes of the gentisic acid cluster were markedly overexpressed in the two *Scedosporium* species analyzed (*Scedosporium apiospermum* and *Scedosporium aurantiacum*). Only the gene encoding a membrane transporter was not overexpressed in the gentisic acid-containing medium. Together, these data suggest the involvement of the lower funneling pathways in *Scedosporium* adaptation to their environment.

## Introduction

Environmental contamination by xenobiotics is now a worldwide phenomenon with frequent serious effects on human and animal health. A large number of fungi characterized as opportunistic pathogens, such as *Exophiala*, *Cladophialophora*, *Aspergillus*, or *Scedosporium* species, are found in human-made environments and exhibit degrading abilities toward aliphatic and aromatic hydrocarbons (Prenafeta-Boldú et al., 2006; Blasi et al., 2016). Interestingly, it has been established for several pathogenic microorganisms also associated with polluted environments that there is a link between their capacity to degrade aromatic pollutants and their virulence. For example, in the bacteria *Acinetobacter baumannii*, the *paaI* and *paaY* genes involved in the catabolism of phenylacetic acid play a role in virulence (Cerqueira et al., 2014). These two genes, as well as six other genes involved in the catabolism of aromatic compounds, are necessary for the multiplication of the bacteria in the *Galleria mellonella* model (Gebhardt et al., 2015). Similarly, in *Escherichia coli*, two clusters of genes involved in the degradation of aromatic compounds (Liu et al., 2015) were not detected in the genome of the non-pathogenic strain PCN061 compared to the genome of the strain PCN031 which is pathogenic for piglets. Regarding fungi, a link between pathogenicity and capacity to degrade the aromatic compounds has been investigated only in *Fusarium oxysporum*, and it was demonstrated that the degradation of aromatic compounds by the *β*-ketoadipate pathway is essential for virulence of this phytopathogenic fungus (Michielse et al., 2012). Considering the low susceptibility to current antifungal drugs of most of the above-mentioned life-threatening fungal pathogens, a better understanding of their adaptative mechanisms to polluted environments could allow to identify interesting metabolic pathways as targets for the development of more potent antifungals.

Lignocellulolytic fungi that are capable to degrade lignin, a polymer of phenylpropanoid units, have received a particular attention during the past two decades since they are also able to efficiently break down synthetic estrogens, polychlorobiphenyl, or emerging micropollutants, such as nanoparticles (Cajthaml, 2015). Recent works evidence that they use a similar enzymatic arsenal to degrade lignin, lignocellulose components, and organic pollutants, such as aromatic hydrocarbons (Korniłłowicz-Kowalska and Rybczyńska, 2015). Lignin degradation by fungi firstly involves extracellular steps leading to various phenolic breakdown products, which are then degraded intracellularly. It may be summarized into two successive processes: (1) formation of a dihydroxylated aromatic ring and (2) ring fission and subsequent reactions channeling to the tricarboxylic acid cycle, through oxaloacetate, fumarate, and pyruvate (Harwood and Parales, 1996). Extracellular oxidative degradation of lignin produces an heterogeneous mixture of aromatic monomers (Bugg et al., 2011) that are further catabolized through the microbial “funneling pathway,” including upper and lower pathways (for a detailed description of the pathways, see Lubbers et al., 2019). In the upper pathways, these aromatic molecules are catabolized into seven main aromatic compounds: hydroxyquinol (benzene-1,2,4-triol), catechol (benzene-1,2-diol), protocatechuate (3,4-dihydroxybenzoic acid), gentisic acid (2,5-dihydroxybenzoic acid), hydroxyquinone (benzene-1,4-diol), gallic acid (3,4,5-trihydroxybenzoic acid), and pyrogallol (benzene-1,2,3-triol; Harwood and Parales, 1996; Johnson and Beckham, 2015; Wang et al., 2017; Lubbers et al., 2019). These compounds serve as substrates in the lower funneling pathways where they undergo a critical ring-opening step which is catalyzed by ring cleavage dioxygenases. While these enzymes have been well studied in bacteria, their fungal counterparts are poorly characterized. To date, four main pathways have been in some extent described in fungi; specific dioxygenases opening the aromatic ring in hydroxyquinone, gallic acid, and pyrogallol have not been yet identified. However, in *Candida parapsilosis*, an alternative pathway was described for hydroxyquinone degradation *via* a hydroxylation step of this molecule by a phenol-2-monooxygenase (Eppink et al., 2000). While gentisic acid undergoes an extradiol (or *meta*)-ring cleavage, hydroxyquinol, catechol, and protocatechuate are converted to 3-oxoadipate/ß-ketoadipate (in a pathway with the same name) through an intradiol or *ortho*-pattern (Harwood and Parales, 1996; Bugg et al., 2011; Lubbers et al., 2019). Finally, genes involved in these lower pathways are usually organized in clusters in the fungal genomes (Greene et al., 2014; Gluck-Thaler et al., 2018).

*Scedosporium* species are soil saprophyte filamentous fungi that may cause in humans a wide variety of infections affecting both immunocompetent and immunocompromised individuals (Cortez et al., 2008). They notably rank second among the filamentous fungi colonizing the lungs of patients with cystic fibrosis (Cimon et al., 2000; Pihet et al., 2009). These fungi are thermotolerant and halophilic and have the ability to survive at very low oxygen pressure and to tolerate high osmotic pressure (Guarro et al., 2006; Cortez et al., 2008). They are mainly found in human-made environments, such as wastewater effluents from sewage treatment plants, urban playgrounds as well as on roadsides, and petrol stations (de Hoog et al., 1994; Kaltseis et al., 2009; Rougeron et al., 2015). Their common occurrence in polluted environments could be related to their ability to grow on gaseous n-alkanes and to use cyclic or aromatic pollutants as carbon and energy sources (April et al., 1998; Claußen and Schmidt, 1998, 1999; Prenafeta-Boldú et al., 2006; Rougeron et al., 2018), using the funneling pathways involved in lignin degradation. Therefore, this work was aimed to characterize *in silico* the lower funneling pathways implemented by the main *Scedosporium* species to degrade lignin and its major degradation intermediates and to validate experimentally part of these bioinformatic results.

## Materials and Methods

### Genome Mining and Phylogenetic Analysis

A literature review was performed to identify reference protein sequences of dioxygenases (notably on KEGG enzyme and UniProt sites). Ortholog protein sequences in the whole genome sequence of the reference strain *Scedosporium apiospermum* Institute of Hygiene and Epidemiology-Mycology (IHEM) 14462 were then searched through tBLASTn analyzes.^1^ Only results with an value of *E* < 1E^−6^ on at least 40% of the query sequence were considered.

The Mega X software (Kumar et al., 2018; Stecher et al., 2020) was used for the phylogenetic study. An alignment was created using ClustalW, and phylogenetic tree was constructed by the maximum likelihood method (Jones et al., 1992).

*Scedosporium* protein sequences were also aligned with bacterial functionally characterized dioxygenases using ClustalW in Geneious software (Kearse et al., 2012).

### Strains

Experiments were conducted on two of the major species within the *Scedosporium* genus: *S. apiospermum* which has been largely studied in our laboratory and commonly occurs in Europe (Kaltseis et al., 2009; Rougeron et al., 2015, 2018), and *Scedosporium aurantiacum* which predominates in Australia (Harun et al., 2010; Rougeron et al., 2018). For each species, three strains were investigated, including both clinical strains deposited at Sciensano (Brussels, Belgium) in the IHEM section culture collection that are publicly available, and clinical or environmental strains that are preserved in our culture collection at Angers University, therefore designated UA: *S. apiospermum* IHEM 14462, IHEM 23580, UA 110350824, and *S. aurantiacum* IHEM 23578, UA 100353192–01, and UA 110344103. The whole genome of all strains of this panel has been sequenced and assembled by our group, but it was annotated only for the reference strain *S. apiospermum* IHEM 14462 (whole genome sequence available in the GenBank database under the accession number JOWA00000000.1; Vandeputte et al., 2014).

All isolates were preserved in our laboratory by freeze-drying. For the experiments, strains were maintained by weekly passages on YPDA plates (containing in g per liter: yeast extract, 5; peptone, 10; dextrose, 20; agar, 20; and chloramphenicol, 0.5) with incubation at 37°C.

### Growth Studies

Growth studies were carried out in triplicate on a synthetic agar-based medium derived from the Scedo-Select III selective culture medium (Pham et al., 2015) and containing in g per liter: carbon source, 0.9; ammonium sulfate, 5; potassium dihydrogenophosphate, 1.25; magnesium sulfate, 0.625; agar, 20; and chloramphenicol, 0.5. A unique carbon source was used in all experiments, either glucose for control conditions, kraft lignin (Sigma-Aldrich), or gentisic acid (Sigma). Inoculation was performed by a central pricking, and growth was evaluated by measuring the diameter of the colonies every day for 10 days. Results were compared with those obtained on YPDA medium.

### RNA Isolation and Reverse Transcription

For RNA extraction, isolates were first grown on potato dextrose agar (Conda, Madrid, Spain) plates at 37°C for 7 days to induce sporulation. Conidia were harvested by aseptically scraping the plates in water and filtrating through Miracloth® mesh filter (Merck, Darmstadt, Germany) to remove the hyphae. Conidia were then enumerated by hemocytometer counts, and 2.10^7^ conidia were inoculated in 50 ml of YEPD liquid medium (containing in g per liter: yeast extract, 5; peptone, 10; dextrose, 20; and chloramphenicol, 0.5). After a 24-h incubation at 37°C with agitation (120 rpm), nascent germ tubes were collected on 11-μm nylon filter, inoculated in 50 ml of derived Scedo-Select III media (containing the same components that the agar-based one except agar) with the appropriate carbon source and incubated with agitation (120 rpm) at 37°C during 4 h. Fungal cells were then collected by filtration through Miracloth® mesh filter, and the fungal material was ground in liquid nitrogen with a mortar and pestle. Total RNAs were extracted using the NucleoSpin® RNA plant kit (Macherey-Nagel, Düren, Germany), according to the manufacturer’s instructions. RNA samples were then treated with 2 U of RNase-free DNase I (Ambion™ Life Technologies, Carlsbad, 168 CA), following the manufacturer’s recommendations. RNA quantity and quality were evaluated by the Qubit assay and electrophoretic analysis. Complementary DNA (cDNA) was synthetized from 500 ng total RNA using High Capacity cDNA Reverse Transcription kit (Applied Biosystems) and random primers, according to the protocol supplied by the manufacturer. After a 10-fold dilution, cDNA samples were used as template for real-time quantitative PCR (qPCR).

### Real-Time Quantitative PCR

Each PCR reaction was performed in a final volume of 12.5 μl containing FAST SYBR®Green PCR Master Mix (Applied Biosystems, Foster City, CA), 200 nM of each primer (Integrated DNA Technologies Inc., Leuven, Belgium), and 2 μl of diluted cDNA. Primers used for qPCR experiments and PCR efficiencies are compiled in Supplementary Table S1. qPCR reactions were carried out on StepOnePlus™ thermocycler (Applied Biosystems) with the following amplification program: 95°C for 20 s, 40 cycles of 95°C for 3 s, and 60°C for 30 s. Melting curve analysis (95°C for 15 s and stepwise annealing from 60 to 95 with 0.3°C increments) was performed immediately after the amplification. For each gene, fold changes relative to standard condition (i.e., in the presence of glucose as carbon source) were calculated with the ∆∆Ct method (Livak and Schmittgen, 2001; Pfaffl, 2001). Two reference genes were selected based on their stable expression whatever the culture conditions and the species considered (validated by an ANOVA statistical test). For each point, three biological replicates and two technical replicates were performed, and a variation in expression of a given gene was considered significant if the Log2 fold change ± standard deviation was >1 or <−1.

## Results

### Bioinformatic Analyses

**Figure 1:**
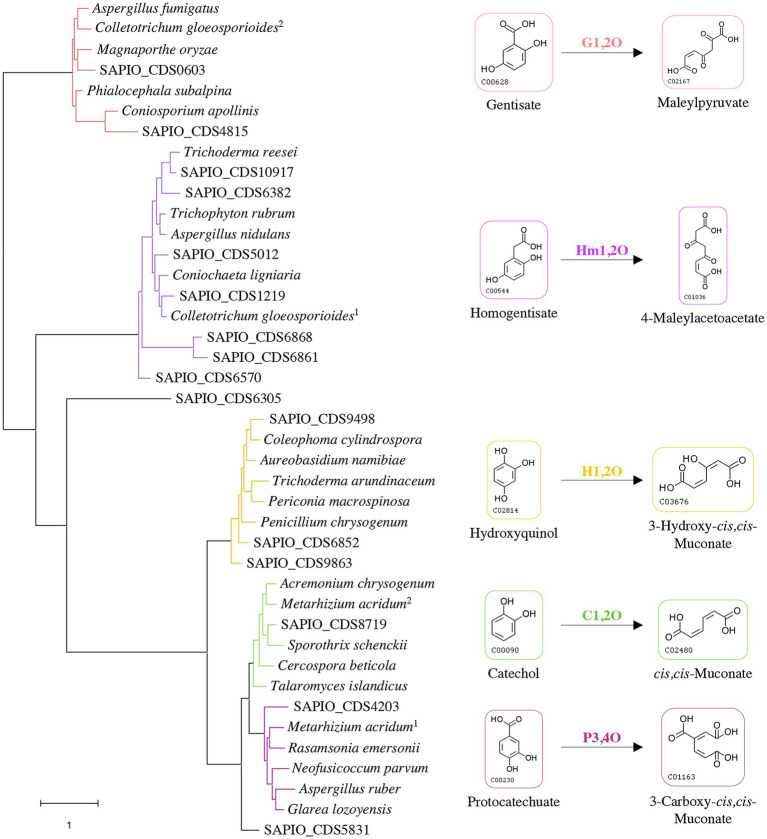
Classification of *Scedosporium* candidate genes among fungal reference sequences of dioxygenases. The evolution history was inferred by using maximum likelihood method and JTT matrix-based model. The tree with the highest log likelihood (−25371.88) is shown. Each color corresponds to a specific dioxygenase: gentisate 1,2-dioxygenase (G1,2O) in red; homogentisate 1,2-dioxygenase (Hm1,2O) in purple; hydroxyquinol 1,2-dioxygenase (H1,2O) in yellow; catechol 1,2-dioxygenase (C1,2O) in green; and protocatechuate 3,4-dioxygenase (P3,4O) in pink. *Scedosporium apiospermum* genes are identified by their accession numbers in the GenBank database, and reference protein sequences used with the corresponding UniProt IDs are the following: *Aspergillus fumigatus* (Q4W9H3); *Colletotrichum gloeosporioides* (^1^: T0JZC0/^2^: T0K095); *Magnaporthe oryzae* (Q2KEQ4); *Phialocephala subalpina* (A0A1L7XPN4); *Coniosporium apollinis* (R7YTB2); *Trichoderma reesei* (G0RIN3); *Trichophyton rubrum* (A0A087PFF1); *Aspergillus nidulans* (Q00667); *Coniochaeta ligniaria* (A0A1J7IAU0); *Coleophoma cylindrospora* (A0A3D8S1I0); *Aureobasidium namibiae* (A0A074WM13); *Trichoderma arundinaceum* (A0A395NVW6); *Periconia macrospinosa* (A0A2V1E4S4); *Penicillium chrysogenum* (B6HA25); *Acremonium chrysogenum* (A0A086T400); *Metarhizium acridum* (^1^: E9DYI0/^2^: E9DYX5); *Sporothrix schenckii* (U7PWF0); *Cercospora beticola* (A0A2S6C3P1); *Talaromyces islandicus* (A0A0U1LSH3); *Rasamsonia emersonii* (A0A0F4YLZ4); *Neofusicoccum parvum* (R1EJ65); *Aspergillus ruber* (A0A017S957); and *Glarea lozoyensis* (S3D670).

Some specific properties of *Scedosporium* species, especially their common occurrence in polluted environments, their ability to grow using lignin as sole carbon source, and their capacity to use aromatic pollutants as carbon and energy sources (April et al., 1998; Claußen and Schmidt, 1998, 1999; Prenafeta-Boldú et al., 2006; Rougeron et al., 2018), suggest the existence of metabolic pathways allowing ring opening of the aromatic compounds that are identical to the lower funneling pathways in the degradation of lignin. Ring-cleaving dioxygenases play a critical role and determine the diversity of lower pathways. Genes reported in the literature as encoding such dioxygenases in fungi were used to screen the genome of the reference strain *S. apiospermum* IHEM 14462 by tBLASTn analyses. Sixteen putative genes were identified. A phylogenetic analysis was carried out to discriminate these enzymes and promote a putative functional association dioxygenase/lower pathway (Figure 1). The coding sequence SAPIO_CDS6305 did not well-align with the other *Scedosporium* coding sequences or the fungal homologues, thus constituting alone a branch of the tree. Two *Scedosporium* proteins were shown to share significant sequence homologies with gentisate 1,2-dioxygenases, and seven with homogentisate 1,2-dioxygenases. Finally, six sequences clustered with intradiol dioxygenases: Three grouped with genes encoding hydroxyquinol 1,2-dioxygenases, one with catechol 1,2-dioxygenase, one with protocatechuate 3,4-dioxygenase, and the last one appeared within intradiol dioxygenases but separated from the three other branches. These results suggest that *S. apiospermum* is able to catabolize the main aromatic intermediates derived from lignin degradation (i.e., gentisic acid, hydroxyquinol, protocatechuate, and catechol), as well as homogentisic acid. However, this last compound is mainly reported in phenylalanine and tyrosine catabolism (Pérez-Pantoja et al., 2008; Perez-Cuesta et al., 2020) as well as in pyomelanin biosynthesis (Mäkelä et al., 2015) and does not seem to be related to lignin degradation, explaining that it was not further investigated here.

**Figure 2:**
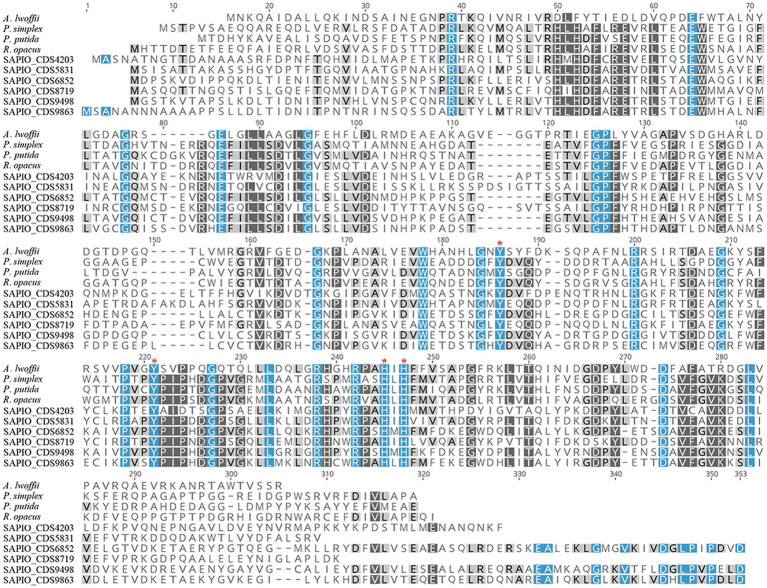
Multiple-sequence alignment of amino acid sequences of *S. apiospermum* intradiol proteins with characterized bacterial dioxygenases of the *ß*-ketoadipate pathway. *S. apiospermum* proteins are identified by their accession numbers in the GenBank database. The bacterial reference protein sequences used, with the corresponding UniProt IDs, are the following: *A. lwoffii* (O33950) from *Acinetobacter lwoffii*; *P. simplex* (Q5PXQ6) from *Pimelobacter simplex*; *P. putida* (C6FI44) from *Pseudomonas putida*; and *R. opacus* (Q6F4M7) from *Rhodococcus opacus* (Semana and Powlowski, 2019).

**Figure 3:**
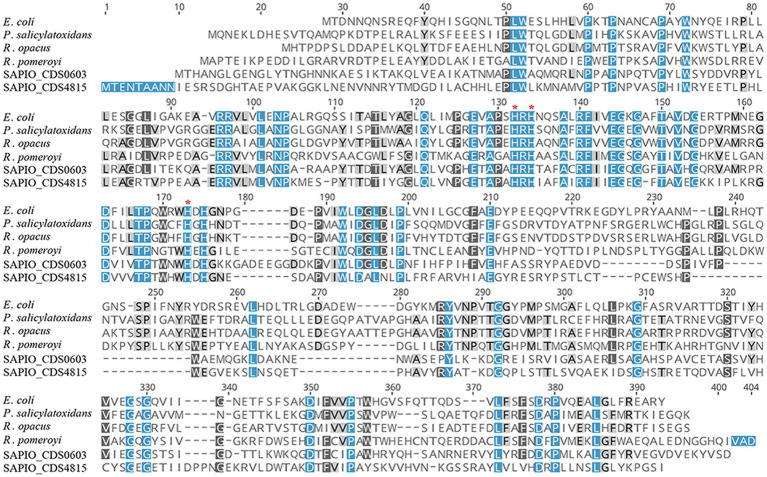
Multiple-sequence alignment of amino acid sequences of *S. apiospermum* dioxygenases with characterized bacterial gentisate 1,2 -dioxygenases. *S. apiospermum* proteins are identified by their accession numbers in the GenBank database. The bacterial reference protein sequences used, with the corresponding UniProt IDs, are the following: *Escherichia coli* (Q8X655) from *E. coli* O157:H7; *P. salicylatoxidans* (Q67FT0) from *Pseudaminobacter salicylatoxidans*; *R. opacus* (Q0PES5) from *Rhodococcus opacus*; and *R. pomeroyi* (Q5LLB1) from *Ruegeria pomeroyi* (Buongiorno and Straganz, 2013; Eppinger et al., 2015).

In order to further characterize the putative *Scedosporium* dioxygenases, a second alignment was performed with bacterial functionally characterized ring-cleaving dioxygenases (i.e., intradiol dioxygenases for SAPIO_CDS9498, SAPIO_CDS6852, SAPIO_CDS9863, SAPIO_CDS5831, SAPIO_CDS8719, and SAPIO_CDS4203, on the one hand, and gentisate 1,2-dioxygenases for SAPIO_CDS0603 and SAPIO_CDS4815, on the other hand). In both cases, a good alignment was observed and multiple conserved residues within the active-sites were shown, as predicted by the CDD (Marchler-Bauer et al., 2015) and CATH (Sillitoe et al., 2021) databases. The conserved residues responsible for active-site nonheme ferric iron coordination and activity in dioxygenases were also conserved in the *Scedosporium* protein sequences: the 4 residues, Tyr(Y)-186, Tyr(Y)-221, His(H)-245, and His(H)-247, within intradiol dioxygenases (Figure 2) and the 3 residues, His(H)-132, His(H)-134, and His(H)-173, within gentisate 1,2-dioxygenases (Figure 3).

**Figure 4:**
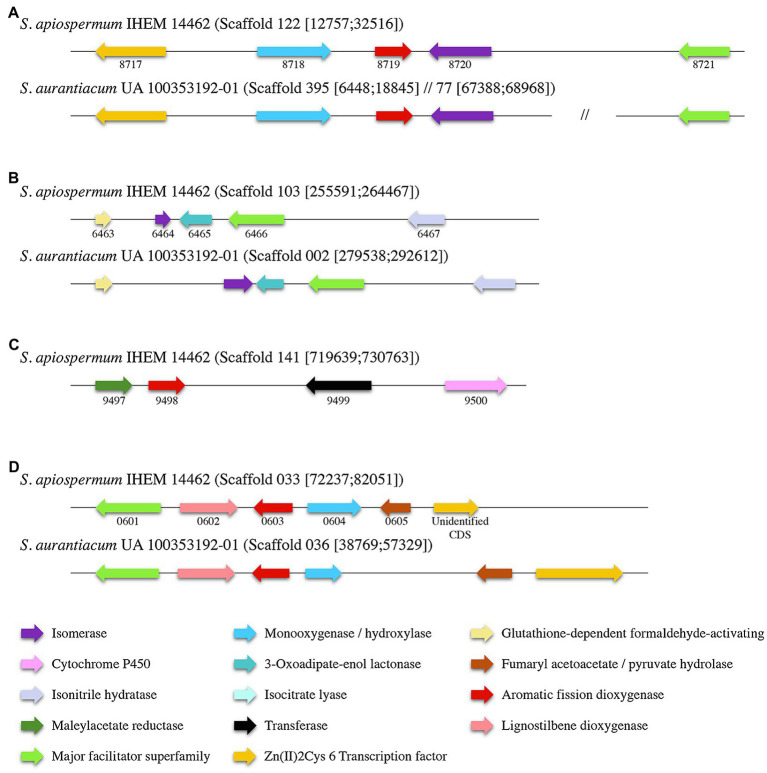
Organization of the gene clusters of the main lower funneling pathways in *Scedosporium* species **(A)**: gene cluster of the catechol degradation pathway; **(B)**: Secondary cluster associated to catechol degradation; **(C)**: gene cluster of the hydroxyquinol degradation pathway; and **(D)**: gene cluster of the gentisic acid degradation pathway. Each color corresponds to a specific enzyme. *S. apiospermum* genes are identified by their CDS accession numbers in the GenBank database.

Identification within the sequences of the putative dioxygenases involved in the lower funneling pathway was carried out by the analysis of the genomic environment of each dioxygenase gene in *S. apiospermum* and *S. aurantiacum* (Figure 4), based on the gene cluster organization described in several ascomycetes by Martins and his colleagues (Martins et al., 2019). In fungi, the protocatechuate pathway is initiated by the intradiol cleavage of protocatechuate that forms 3-carboxy-cis,cis-muconate. This product is then converted into 3-carboxymuconolactone and 3-oxoadipate. In *Aspergillus nidulans*, three genes were assigned to this pathway: an intradiol dioxygenase, a 3-carboxy-*cis,cis*-muconate cyclase and a 3-carboxymuconolactone hydrolase. In *Scedosporium* genome, SAPIO_CDS4203 is the only gene identified as encoding a protocatechuate 3,4-dioxygenase. Moreover, orthologs of the two other essential genes involved in protocatechuate catabolism in *A. nidulans* were identified in *S. apiospermum* genome, located on distinct scaffolds SAPIO_CDS4774/SAPIO_CDS8203 for the 3-carboxy-*cis,cis*-muconate cyclase and SAPIO_CDS10656 for the 3-carboxymuconolactone hydrolase. As previously reported in *A. nidulans* (Martins et al., 2015), genes involved in the intradiol cleavage of protocatechuate were not clustered in *Scedosporium* genome. Conversely, the catechol 1,2-dioxygenase SAPIO_CDS8719 was localized within a cluster of five genes (SAPIO_CDS8717 to SAPIO_CDS8721) involved in phenol or catechol catabolism (Figure 4A), which comprises (1) a fungal transcription factor (SAPIO_CDS8717); (2) a phenol 2-monooxygenase (SAPIO_CDS8718), structurally related to 4-hydroxybenzoate 3-hydroxylase from the ortho-hydroxylases clade (Westphal et al., 2021) which suggests its involvement in conversion of *p*-hydroxybenzoic acid molecules (Supplementary Figure S1); (3) a muconate cycloisomerase (SAPIO_CDS8720), the second key enzyme of this degradation pathway (Martins et al., 2019); and (4) a membrane transporter belonging to the major facilitator superfamily (MFS; SAPIO_CDS8721). In *A. nidulans*, two additional genes encoding a muconolactone isomerase (AN4061) and a 3-oxoadipate-enol lactonase (AN4531) have been reported as essential for the degradation of catechol (Martins et al., 2019). Interestingly, orthologs of these genes, SAPIO_CDS6464 and SAPIO_CDS6465, respectively, were identified in another scaffold (scaffold 103) of the S. apiospermum genome (Figure 4B), clustered with a MFS gene (SAPIO_CDS6466) and genes encoding for proteins involved in the early stages of formaldehyde detoxification (SAPIO_CDS6463 and SAPIO_CDS6467). Moreover, many aromatic compounds are channeled to the hydroxyquinol pathway (such as *p*-hydroxybenzoic and vanillic acids). This is a very short pathway, comprising only one specific dioxygenase and a maleylacetate reductase. In *S. apiospermum* genome, these genes are adjacent in scaffold 141 (SAPIO_CDS9497 and SAPIO_CDS9498 encoding a maleylacetate reductase and the dioxygenase, respectively) followed by genes encoding a CoA-transferase (SAPIO_CDS9499) and a cytochrome P450 (SAPIO_CDS9500; Figure 4C). Finally, the gentisic acid derives from 3-hydroxybenzoic acid and *p*-hydroxybenzoic and salicylic acids. The cluster organization of the gentisic acid pathway in *Scedosporium* genome was previously reported (Rougeron et al., 2018; Martins et al., 2019), but the fine composition of this cluster diverges between the two studies: The former included two supplemental genes within the cluster (i.e., a gene encoding a quinone oxidoreductase – SAPIO_CDS0606 – and a not annotated sequence encoding a Zn(II)2Cys6 transcription factor). As a gene encoding Zn(II)2Cys6 transcription factor is commonly observed in fungal gentisic acid pathway (Martins et al., 2019), the not annotated sequence encoding this protein was conserved (unidentified CDS). Conversely, the gene encoding the quinone oxidoreductase (SAPIO_CDS0606) was excluded from the gene cluster. Consequently, we define here that the gentisic acid pathway comprised six genes encoding: an MFS (SAPIO_CDS0601), a cytochrome P450 (SAPIO_CDS0602), the specific dioxygenase (SAPIO_CDS0603), a monooxygenase (SAPIO_CDS0604), a fumaryl acetoacetate hydrolase (SAPIO_CDS0605), and the Zn(II)2Cys6 transcription factor (unidentified CDS; Figure 4D). The monooxygenase gene that was structurally related to 4-hydroxybenzoate 1-hydroxylases from the decarboxylating hydroxylases clade (Westphal et al., 2021) suggesting an involvement in conversion of *p*-hydroxybenzoic acid molecules (Supplementary Figure S1) was shorter in *S. aurantiacum,* one of the three FAD fingerprints being missing (Supplementary Figure S2). Similar gene organization was found in *S. aurantiacum* genome except for the hydroxyquinol pathway, since we did not find any orthologs of these corresponding genes.

Based on these *in silico* analysis, we identify the most probable ring-cleaving dioxygenases involved in the lower funneling pathways among 16 candidate genes.

### Validation of the Gentisic Acid Cluster

**Figure 5:**
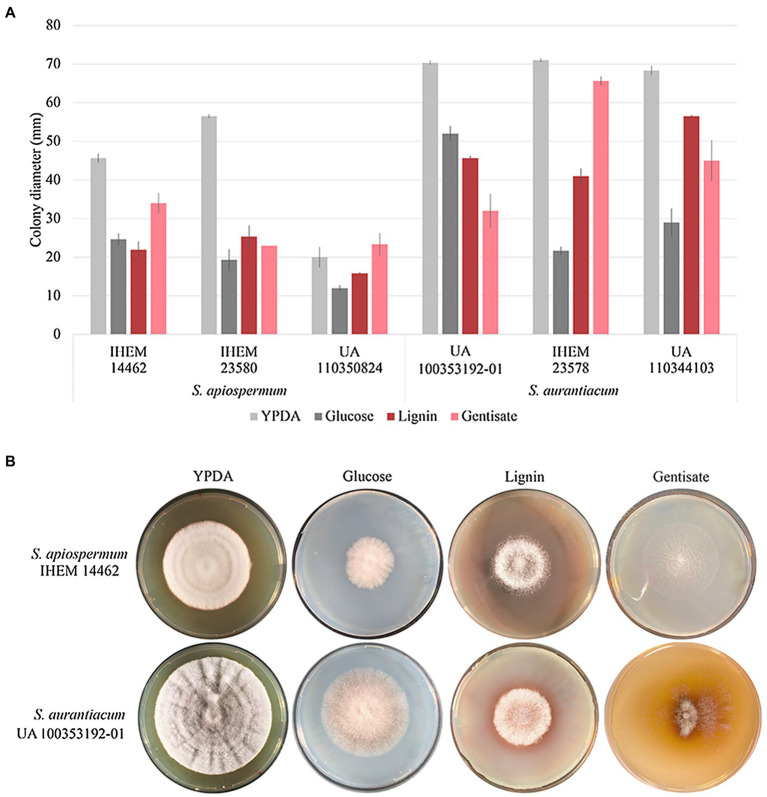
Influence of the carbon source on growth of *Scedosporium* isolates. Clinical (deposited at Sciensano in Brussels, Belgium, and designated by IHEM for Institute of Hygiene and Epidemiology-Mycology section culture collection) as well as clinical or environmental (preserved in our culture collection at the University of Angers and designated UA) strains of *S. apiospermum* and *S. aurantiacum* were cultivated in triplicate on YPDA or lignin or gentisic acid-containing agar-based media for 10 days at 37°C. **(A)**: The growth was evaluated by measuring the diameter of the colonies. Bars indicate the standard deviation of the mean. **(B)**: Aspect of the colony at day 10.

In order to validate our bioinformatic results, we focus on the gentisic acid pathway. Growth of the two *Scedosporium* species (*i.e.*, *S. apiospermum* and *S. aurantiacum*) was investigated on synthetic agar-based media containing lignin or gentisic acid as the sole carbon source, or glucose for control conditions. Figure 5 illustrates the results obtained after 10 days of incubation for carbon source and a representative strain of each species. Even if growth was reduced compared to YPDA conditions, all isolates were able to use lignin or gentisic acid as carbon and energy source.

**Figure 6:**
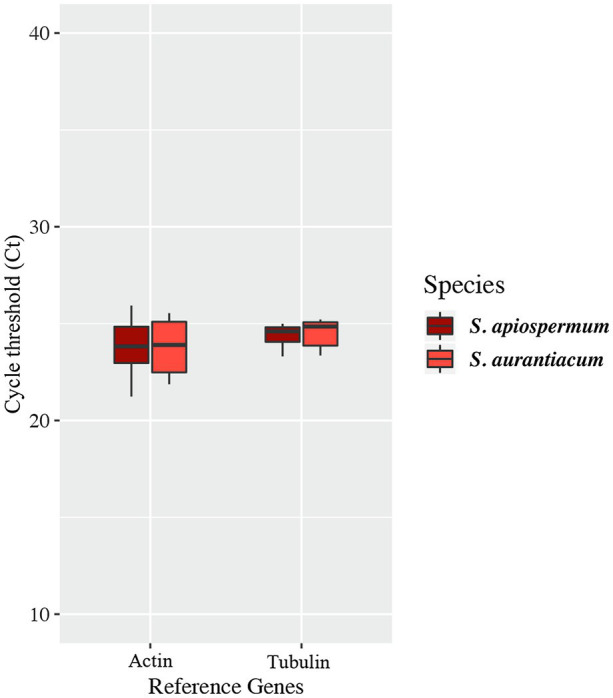
Variations of expression of reference genes in the different culture conditions studied. Central lines in each box indicate the median, and the lower and upper rims represent the first and third quartiles. The whiskers extend to the lowest and highest datum within 1.5-fold the interquartile range from the lower or upper quartile.

**Figure 7:**
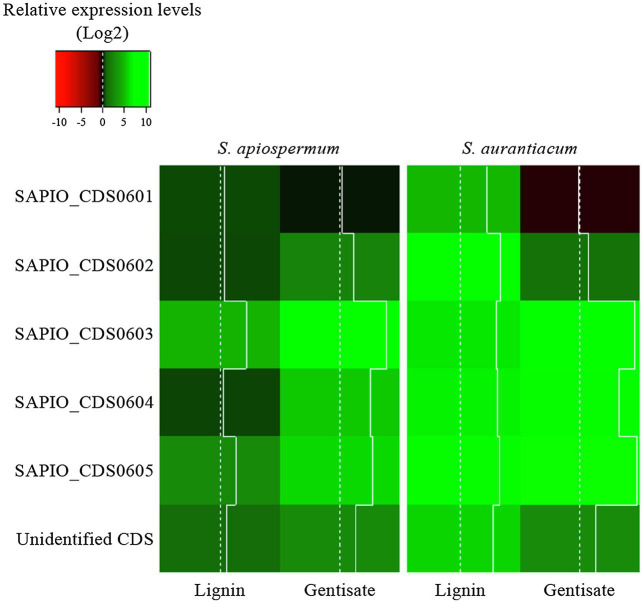
Relative expression levels (Log2) of genes of the gentisic acid degradation pathway in the fungus grown in the presence of lignin or gentisic acid. *S. apiospermum* or *S. aurantiacum* strains were cultivated in a lignin- or gentisic acid-containing liquid culture medium for 4 h at 37°C, and the expression level of the genes (designated by their GenBank accession number for *S. apiospermum*, or their orthologs in *S. aurantiacum* genome) was evaluated by quantitative PCR after reverse transcription. For each species, results correspond to an average of real-time qPCR analysis performed on three different isolates.

In order to follow the relative expression of the genes of the gentisic acid pathway according to the ∆∆Ct method, two reference genes were validated in our experimental conditions. Thus, actin and *β*-tubulin genes were used to standardize the expression of the target genes (Figure 6). In both media, a similar pattern was observed for *S. apiospermum* and *S. aurantiacum*. In gentisic acid-containing medium, apart from the MFS gene SAPIO_CDS0601, all the genes of this cluster showed a marked overexpression compared to growth control conditions. In lignin-containing medium, all the genes were overexpressed. However, discrepancies between the two species were observed in the gene expression levels. All the genes were strongly overexpressed in *S. aurantiacum* (between 27-fold increase for SAPIO_CDS0601 and 161-fold increase for SAPIO_CDS0602), whereas a 8-fold increase only was seen for *S. apiospermum* in the expression of SAPIO_CDS0601, SAPIO_CDS0602, and SAPIO_CDS0604. Only the gentisate 1,2-dioxygenase SAPIO_CDS0603 and the fumaryl pyruvate hydrolase SAPIO_CDS0605 were markedly overexpressed (136- and 37-fold increase, respectively; Figure 7). These results demonstrated that expression of the genes of this cluster was induced by the presence in the medium of gentisic acid but also of lignin.

## Discussion

Although it is well established that the ecology of *Scedosporium* species is associated with human activities, there is still controversy regarding the natural habitat of these fungi. Nevertheless, there is now an increasing body of evidence that these soil fungi have lignocellulolytic properties. Several strains of the genus *Scedosporium* were recovered from submerged woods in estuarian (Kirk, 1967) or marine coasts (Azevedo et al., 2011), and an enrichment procedure was developed for the recovery of these fungi from liquid samples using wooden blocks which showed abundant development of ascomata on the wood support (Kirk, 1967). Likewise, several strains have been reported from forest soils or wood (de Hoog et al., 1994; Gilgado et al., 2005), but also from xylophagous insects, including Amazonian *Nasutermites* sp. termite workers (Nirma et al., 2013), Chinese bark beetles (Wu et al., 2014), and larvae of some wood-feeding Coleoptera collected in tropical forests of Costa Rica (Rojas-Jiménez and Hernández, 2015). Besides, these last strains also showed capacity to degrade the main structural components of wood, i.e., cellulose, lignin, *β*-D-xylan, *β*-D-cellobiose, and *β*-D-glucan (Rojas-Jiménez and Hernández, 2015). Finally, in two recently published metagenomic studies aimed to investigate the effects of copper-based fungicides used for wood protection (Lasota et al., 2018), or to analyze the fungal community structure and its association with the cause of decay on the wooden pillars of an ancient archway in Beijing (China; Ma et al., 2020), the genus *Scedosporium* was revealed among the main fungal operational taxonomic units identified. Moreover, *Scedosporium* species are able to grow using lignin as a carbon source, and the semi-selective medium we developed in the laboratory for *Scedosporium* isolation relies on the ability of the fungus to use 4-hydroxybenzoate as a carbon source. The bioinformatic part of this study confirmed that the *Scedosporium* genome comprises all the genes required for the main lower funneling pathways involved in the catabolism of aromatic intermediates derived from lignin degradation: the three branches of the 3-oxoadipate pathway (catechol, hydroxyquinol, and protocatechuate branches) and the gentisic acid pathway. Apart from the protocatechuate branch of the 3-oxoadipate pathway, all these genes are organized in clusters. Considering the protocatechuate degradation, orthologs of three essential genes encoding a protocatechuate 3,4-dioxygenase, a 3-carboxy-*cis,cis*-muconatecyclase and a hydrolase with a decarboxylase activity were identified as described in *A. nidulans*, suggesting functionality of the pathway. Likewise, our experiments validated the functional character of the gentisic acid pathway and its activation when *Scedosporium* is cultivated in the presence of lignin. Further experiments are required to validate the other lower pathways. Moreover, biochemical analyses are required to evidence lignin degradation and to characterize the involvement of these funneling pathways in this degradation.

Interestingly, these central metabolites derived from lignin are also the result of the degradation of many other aromatic molecules, such as certain environmental pollutants and some plant defense toxins (flavonoids and stilbenes; Naoumkina et al., 2010; Enguita and Leitão, 2013; Wang et al., 2013; Greene et al., 2014). Therefore, the presence of all the genes of the lower funneling pathways in the genome of *Scedosporium* species may partly explain, for example, their ability to degrade *p*-cresol (Claußen and Schmidt, 1998, 1999) and their bleaching properties (Tigini et al., 2014). *Scedosporium* abundance in human-made or polluted environments correlated to the identification of these lower pathways within their genome suggests that *Scedosporium* species are active elements of ecological recycling and may play an important role in agriculture. Although the opportunist pathogen character may impede the use of *Scedosporium* in bioremediation, the enzymatic arsenal of these fungi could be of industrial interest.

Moreover, a link between aromatic hydrocarbon catabolism and pathogenicity for human and animals has been suggested. Adaptation of *Scedosporium* species to extreme environments, as well as their thermotolerance and their melanized spores, may predispose these fungi toward pathogenesis favoring their survival in growth factor and nutrient-limited microenvironment encountered in the host tissues. Recently, it was demonstrated that the exposure to an aromatic chlorinated compound and its degradation induce an increased pathogenic potential of fungal communities, which in turn may increase the dispersal of airborne opportunistic pathogens (Martins et al., 2018). As already mentioned, a link between pathogeny and degradation of aromatic compounds has been suggested, but regarding fungi, it was actually demonstrated only for phytopathogenic ones. The ability of these fungi to metabolize antifungal defense components of the plant host appears as an important virulence factor. For example, *Sclerotinia sclerotiorum* metabolizes salicylate, a key defense-signaling molecule (Penn and Daniel, 2013), and the virulence of *Botrytis cinerea* on grape is correlated at least in part with its ability to metabolize stilbene-type phytoalexins (Sbaghi et al., 1996). Involvement of these lower funneling pathways in the pathogenesis of *Scedosporium* species should be further analyzed (pathway silencing by dioxygenase invalidation and analysis of virulence of deficient mutants in mouse model) in order to identify new therapeutic targets for drug design.

## Data Availability Statement

Publicly available datasets were analyzed in this study. This data can be found here: JOWA01000000 for *Scedosporium apiospermum* genome and JUDQ01 for *Scedosporium aurantiacum* genome.

## Author Contributions

WP performed the experiments of the initial manuscript and data analysis, and wrote the first draft. KR participated to the revision work. J-PB provided the funding for research and reviewed the manuscript. SG managed the study and wrote the final manuscript. All authors contributed to the article and approved the submitted version.

### Conflict of Interest

The authors declare that the research was conducted in the absence of any commercial or financial relationships that could be construed as a potential conflict of interest.

## Footnotes

^1^https://blast.ncbi.nlm.nih.gov/Blast.cgi
