# Supplementary material for: Lower Funneling Pathways in Scedosporium Species
Source: Front Microbiol. 2021 Jul 2;12:630753. doi: 10.3389/fmicb.2021.630753 (PMC8283699; doi:10.3389/fmicb.2021.630753)

Table S1: Primers used in qPCR experiment for gentisate cluster expressions.

Gene	Primers	Efficiency (%)
**SAPIO_CDS0601**	F : GTCTGATCATTCGAAACCAC<br>R : CAGGATAGCAATCAAGAAGG	100,336
**Ortholog in *S. aurantiacum* (Scaffold 036 [38769 ; 40741])**	F : TCGCAATCACTTACCTTCTT<br>R : GCGACCACCATAAACATAAT	115,543
**SAPIO_CDS0602**	F : CGTTCTACAGGGTCATGC<br>R : CAGAGCCCTATTGTCTCAAG	81,726
**Ortholog in *S. aurantiacum* (Scaffold 036 [42636 ; 44423])**	F : CGTTCTACAGGGTCATGC<br>R : ATCTTGAACTCCACGGCATC	100,98
**SAPIO_CDS0603**	F : CCTTTGGTCCTATGATGATG<br>R : AGAGTGTCGGTTGTGTAAGG	98,675
**Ortholog in *S. aurantiacum* (Scaffold 036 [44945 ; 46117])**	F : TTATCCACTTCCCGATACAC<br>R : GGACGCAGTTTCACAGATAG	97,149
**SAPIO_CDS0604**	F : GGCGTGGCTACTTGAGAAAC<br>R : TGGGACAAGACCATGTTGAA	93,526
**Ortholog in *S. aurantiacum* (Scaffold 036 [46611 ; 47746])**	F : GAAGCAATTGGGTACACG<br>R : CCAGCAATGCAGTAGGTC	102,964
**SAPIO_CDS0605**	F : GGTCTAAAGGCCAAGGTTAT<br>R : ACTACGTTGGTATCGTGGTC	81,98
**Ortholog in *S. aurantiacum* (Scaffold 036 [51932 ; 53046])**	F : GGATGATTTGGTCTTTTCTG<br>R : CTTCACTTCCATCTTTGTCC	94,038
**Unidentified CDS**	F : TACGAACTGCCTGAAACATC<br>R : GAGAGACGCTGATGATGC	98,577
**Ortholog in *S. aurantiacum* (Scaffold 036 [53819 ; 56529])**		90,403

qPCR: quantitative polymerase chain reaction; SAPIO_CDS: Coding sequences of *Scedosporium apiospermum*; F: Forward; R: Reverse.

Figure S1 : Classification of *Scedosporium* monooxygenases genes among microbial reference sequences of monooxygenase

Scedosporium apiospermum genes are identified by their Genbank accession numbers and reference protein sequences used with the corresponding UniProt IDs are the following : *Candida parapsilosis* (1: G8BGH1 / 2: G8B709); *Trichosporon cutaneum* (P15245); *Comamonas testosteroni* (1: Q6SSJ6 / 2: A0A1V0J9R0); Aspergillus niger (G3Y748); *Pseudomonas putida* (Q59713); Agaricus bisporus (Q92402); *Boreostereum vibrans* (A0A167KUL3); *Pseudomonas fluorescens* (P86491); *Staphylococcus albus* (L7PLD1); *Rhodococcus jostii* (Q0SFK6) (Westphal *et al.,* 2021).

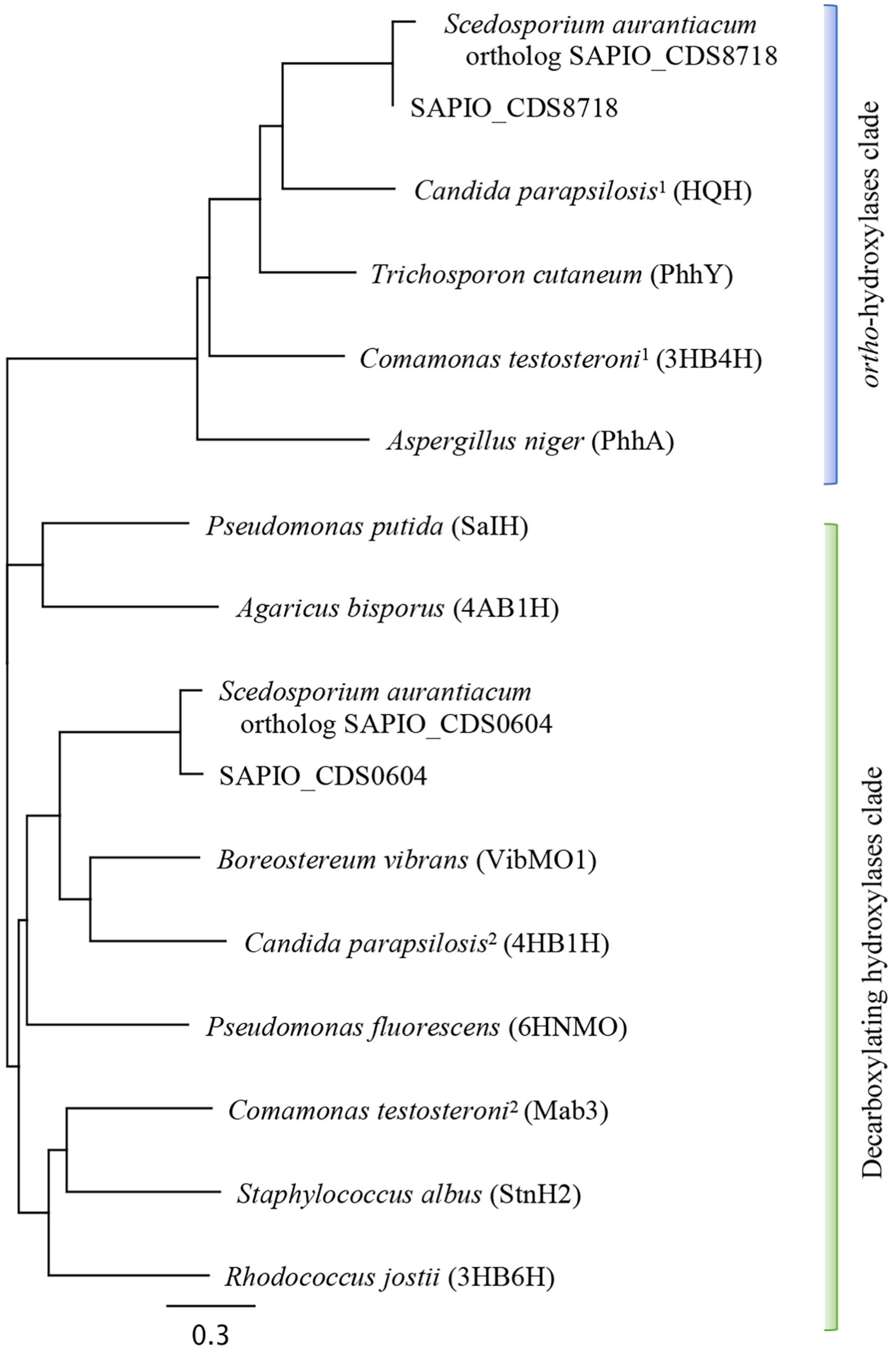

Figure S2: Multiple-sequence alignment of amino acid sequences of *S. apiospermum* monooxygenase SAPIO_CDS0604 and ortholog in *S. aurantiacum* with microbial reference sequences of 4-hydroxybenzoate 1-hydroxylases

FAD binding-domains are marked in yellow and active site residues in red. The reference protein sequences used with the corresponding Uniprot IDs are the following : *C. parapsilosis* (G8B709) from *Candida parapsilosis* and *R. jostii* (Q0SFK6) from *Rhodococcus jostii* (Westphal *et al*., 2021).

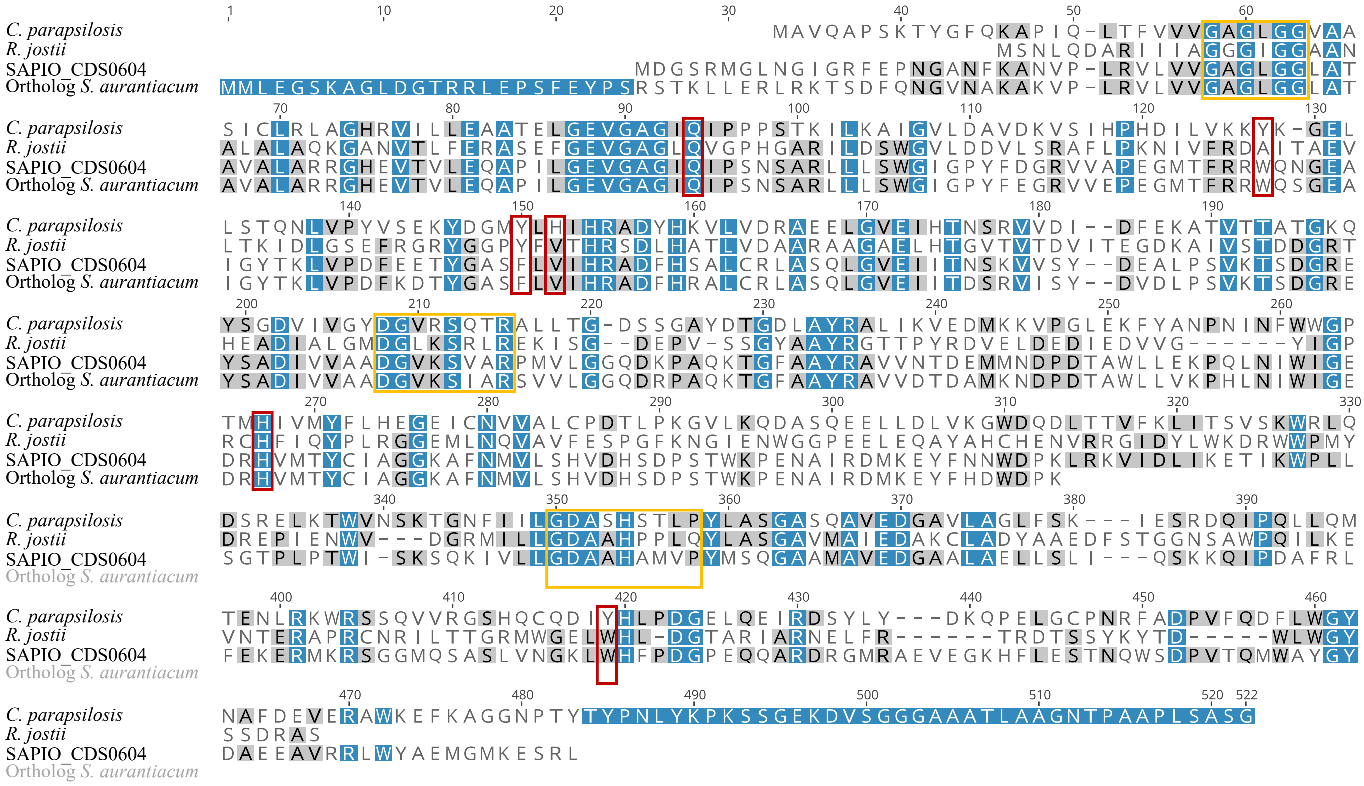

Supplement: Supplementary file 1 [file Data_Sheet_1.PDF]
